# The Sufferings of Christ

**Published:** 1887-11-19

**Authors:** 


### WORDS OF CONSOLATION.

[Communications for this column should be addressed "Chaplain," care of the Editor of THE HOSPITAL, who will gratefully welcome any suggestions or inquiries from matrons, nurses, and the staff generally.]

### LIX.—THE SUFFERINGS OF CHRIST.

(*For Reading to the Sick.*)

PILATE took Jesus and scourged Him (John xix. 1). "This act of Pilate," says St. Augustin, "we must suppose was for no other reason but that the Jews might be satiated with His sufferings, and so desist from raging for His death." And again, the same author says: "That His enemies might be satisfied with their mockeries and not thirst for His blood." And Origen says in like manner: "That when they saw what the soldiers had done, they might pause a little from their passion, therefore he led Him forth thus crowned." Thus, while the unjust judge has not courage to acquit Him whom he pronounces innocent, even his compassions only serve to add to the afflictions and sorrows of Christ. Besides the actual scourging, it appears that our Lord suffered grievously from buffeting and blows, and from the roughest possible handling. "With His stripes we are healed." (Isaiah liii. 5). But what is the connexion between the stripes and the healing? Think for a moment how much brutality on man's part may have been atoned for here, how much wanton cruelty, our cruelty to one another, or to the lower creatures, our unkindness, especially our sins of violence. Every time a blow has been needlessly or wantonly inflicted on one of God's creatures, an atonement has been called for. Every stripe unjustly given, whether by some miserable coward with a stick or stave, or with the palm of the hand by an angry parent, all the rude buffeting from which the meek of the earth and little children and innocent animals have recoiled, all such cruelties are brought to mind in this portion of the sacrifice. You have only to consider the fearful amount of wickedness of this kind for ever going on in the world so loudly alling for atonement, and you may see at once why such prominence is given to the stripes inflicted upon our Lord. But this is also recorded, that we may have the best possible motive supplied us for never again ill-treating a fellow-creature. We are only healed by His stripes in so far as they breed in us a deadly hatred of cruelty in every form, and a determination to put a stop to it, or at least to protest against it, whenever the opportunity occurs. It is terrible to think how many people in this professedly Christian country have their hearts so steeled, that although they may never be guilty of actual cruelty or violence themselves will nevertheless decline to lift their little finger to stay iniquity of this kind, be it ever so close to their door. Happy are those whose hearts are tender enough to be pained at the sight of the smallest unnecessary infliction of violence. They cannot endure preventible suffering; for "with His stripes they are healed."

Otherwise, what is the retribution pointed to for people who have known of His stripes, and yet persisted, until they have earned a character for violence, or unkindness, or for some kind of ill-treatment which causes heartaching? Let them remember there is the being beaten with many stripes, or few, according to the knowledge with which the sin has been committed (Luke xii. 47, 48). We do not plead for a literal interpretation here necessarily; but the analogy is at least curious as it is awful to contemplate. And this is more especially to be noticed when it is considered that the words were uttered in connexion with a parable concerning one who was guilty of violence, and said in his heart, "My Lord delayeth his coming," and began to beat the menservants and maidens who were in his employment.

